# Visualization and quantification of 4D blood flow distribution and energetics in the right ventricle

**DOI:** 10.1186/1532-429X-13-S1-O83

**Published:** 2011-02-02

**Authors:** Carl Johan Carlhäll, Alexandru G Fredriksson, Jakub Zajac, Jonatan Eriksson, Petter Dyverfeldt1, Jan Engvall, Ann F Bolger, Tino Ebbers

**Affiliations:** 1 Center for Medical Image Science and Visualization (CMIV), Linköping University, Linköping, Sweden; 2 University of California San Francisco, San Francisco, CA, USA

## Background

Right ventricular (RV) function has important prognostic value in both right- and left-sided acquired and congenital heart diseases (1). Assessment of RV function is challenging because of its complex crescent shaped geometry and load conditions being significantly influenced by respiration. Incremental insights into RV blood flow patterns have the potential to add to our understanding of RV function (2), but remain incompletely characterized.

## Hypothesis

We hypothesized that a specific portion of the RV end-diastolic (ED) blood volume is prepared for effective systolic ejection and that this sub-volume can be identified based on its pre-systolic kinetic energy (KE) and location.

## Method

**Figure 1:**
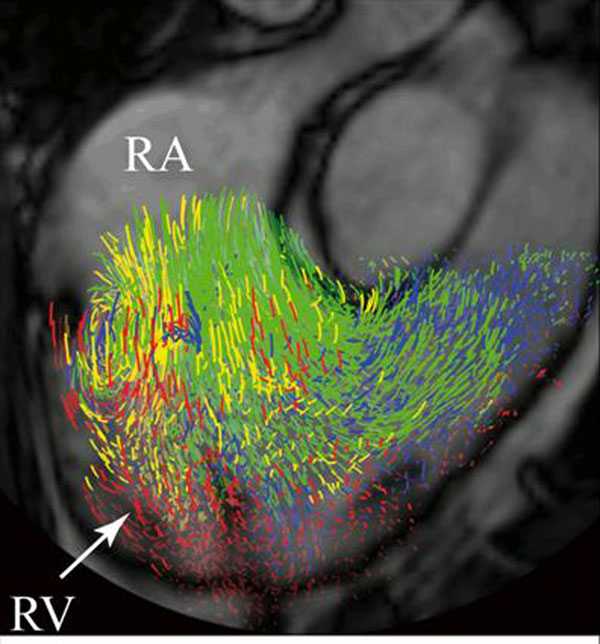
Visualization of right ventricular (RV) flow at end-diastole in a healthy subject. *Direct Flow* (green), blood that enters and leaves the RV within the analyzed cardiac cycle (CC), *Retained Inflow* (yellow), blood that enters but does not leave the RV within the analyzed CC; *Delayed Ejection Flow* (blue), blood that leaves but does not enter the RV within the analyzed CC. RA, right atrium; RV, right ventricle.

**Figure 2:**
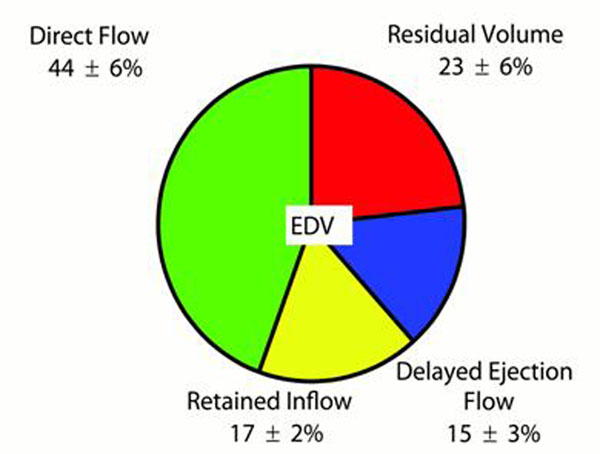
Illustration of the four right ventricular (RV) flow components as a percentage of RV end-diastolic volume (EDV) in healthy subjects.

Three-directional, 3-dimensional cine phase-contrast CMR velocity-data and morphological bSSFP images were acquired on a 1.5T scanner (Philips Achieva) in ten healthy subjects (4 female, aged 46±17 years [mean±SD]). A previously validated method was used for the flow analysis (3): The RV endocardium was segmented (http://segment.heiberg.se) from the morphological images at ED and end-systole (ES). Pathlines were emitted from the ED blood volume and traced forward and backward in time until ES. Accordingly, the ED blood volume could be automatically separated into four functional flow components (3,4)(Figures 1 and 2). By the volume occupied by each trace, its velocity and the density of blood, the kinetic KE was calculated throughout the cardiac cycle.

## Results

**Figure 3:**
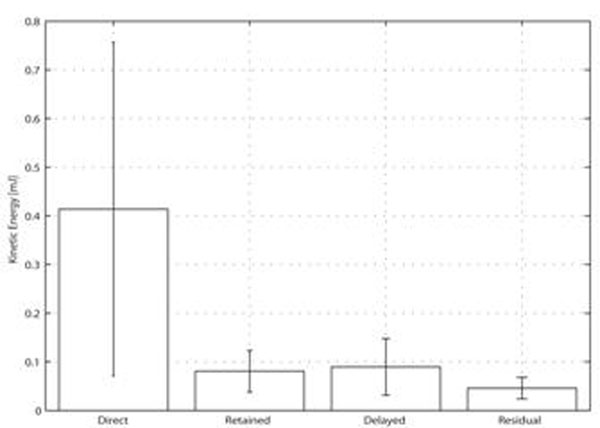
End-diastolic kinetic energy for the four right ventricular flow components in healthy subjects.

The portion of RV inflow passing directly to outflow (*Direct Flow*), possessed a larger pre-systolic KE than the other three flow components (p<0.01)(Figure 3). The *Direct Flow* had a larger volume than the other flow components (p<0.001), and was located mainly in the basal half of the ventricle (Figures 1 and 2). The *Residual Volume* was larger than the *Delayed Ejection Flow* (p<0.01) and the *Retained Inflow* (p<0.05), and was located mainly in the apical half of the ventricle (Figures 1 and 2).

## Conclusions

Semi-automatic analysis of 4D CMR velocity data allows separation of RV flow into distinct functional components. The present findings suggest that diastolic flow in the normal RV creates favorable conditions for effective systolic ejection, defined by pre-systolic KE and location, for the *Direct Flow* component. These flow-specific aspects of RV diastolic-systolic coupling may provide new useful perspectives on RV- and interventricular function in health and disease.
